# Route selection impairment and microglia activation in a rodent model of attention-deficit hyperactivity disorder

**DOI:** 10.3389/fncel.2026.1797607

**Published:** 2026-05-05

**Authors:** Sophia G. Skubic, Zoe Wynter, Madeline M. Kramer, Jena B. Hales

**Affiliations:** Department of Neuroscience, Cognition and Behavior, University of San Diego, San Diego, CA, United States

**Keywords:** ADHD, microglia, neuroinflammation, rodent model, route selection, traveling salesperson problem

## Abstract

**Introduction:**

The ability to remember locations of objects and make spatial decisions is critical when navigating an environment. Such tasks, however, can be difficult for individuals with certain neurological conditions, such as attention deficit hyperactivity disorder (ADHD). The traveling salesperson problem (TSP), a naturalistic spatial foraging task, has been effectively used to examine these spatial navigational processes in rodents, as this optimization task requires subjects to identify the shortest route of travel between a certain number of targets in an open arena. Previous studies using the TSP task have found that rats with hippocampal or medial entorhinal cortex lesions are impaired on measures of spatial memory, but not spatial decision-making or route selection.

**Methods:**

The current study examined the performance of male and female spontaneously hypertensive rats (SHR), the most widely used rodent model of ADHD, relative to their control model, Wistar Kyoto (WKY) rats, on the TSP task.

**Results:**

Our behavioral findings suggest that both male and female SHRs have greater deficits in route selection compared to WKY rats, but they show intact performance on measures of spatial memory. We also examined microglia expression as a marker of neuroinflammation in the prefrontal cortex, hippocampus, and medial entorhinal cortex. SHRs had a greater percentage of hypertrophic microglia, indicating extended periods of inflammation or activation, in the infralimbic area of the prefrontal cortex and in the dentate gyrus. Within the dentate gyrus, the female SHRs showed an increased percentage of hypertrophic microglia compared to female WKY rats.

**Discussion:**

This study expands on existing literature within ADHD-model male and female rats by exploring their ability to effectively route optimize using a naturalistic task.

## Introduction

To successfully navigate the environment, individuals rely on the critical ability to remember the spatial locations of objects and to make navigational decisions based on that information. These common everyday activities are cognitively complex and dynamic, requiring a combination of spatial working memory, route selection, and decision-making. Unfortunately, these tasks can be considerably more difficult for individuals with certain neurological conditions or disorders, including attention deficit hyperactivity disorder (ADHD) (Del Lucchese et al., 2021). ADHD is a complex behavioral disorder that can lead to organizational, focus-related, and decision-making difficulties (American Psychological Association, 2023), along with deficits in spatial working memory (Raiker et al., 2012). Effective research into the neurological mechanisms that underlie these complex cognitive processes is essential for developing a better understanding of the causes and potential treatments of these disorders, and animal models can provide critical insights.

The spontaneously hypertensive rat (SHR) is the most widely investigated animal model of ADHD to date. Derived from the Wistar-Kyoto (WKY) rat (Okamoto and Aoki, 1963), the SHR displays many of the major behavioral characteristics of ADHD, including inattentiveness, hyperactivity, impulsivity, and some genetic and brain structure similarities to people with ADHD (Sagvolden, 2000). For example, both SHRs and humans with ADHD show variations in the dopamine active transporter 1 (DAT1) gene, which regulates dopamine levels in the brain (Mill et al., 2005). In addition, structural imaging studies have shown reduced basal ganglia volumes in both SHRs and humans with ADHD (Hsu et al., 2010; Qiu et al., 2009). Although ADHD humans typically show impairments in working memory (Alderson et al., 2013; Frazier et al., 2004), studies examining working memory in SHRs have yielded inconsistent results. Some studies have reported SHR deficits in working memory and learning (Meneses et al., 1996; Mori and Van Zijl, 1995; Nakamura-Palacios et al., 1996; Tchekalarova et al., 2023; Wyss et al., 1992), while others have found intact working memory when controlling for locomotor function (Sontag et al., 2013) or deficits in other aspects of executive functioning but not working memory (Clements and Wainwright, 2006; Robertson et al., 2008). A recent study examined SHR performance on two hippocampus-dependent memory tasks and found deficits in delay-dependent working memory and elapsed time processing (Anderson et al., 2024). In addition to measuring hippocampus-dependent memory deficits in SHRs, that study also investigated the role of neuroinflammatory signaling and examined sex as a biological variable. Modest differences were found between strains and sexes in hippocampal cytokine expression (specifically TNF-ɑ, IL-4, IL-10, and IL-18). For TNF-ɑ expression in the ventral hippocampus, WKY female rats had a higher concentration than the other three groups (WKY males, SHR females, and SHR males). For IL-4 and IL-10 expression in the ventral hippocampus, WKY males had a higher concentration than the other three groups. For IL-18 expression in the dorsal hippocampus, SHR females had a lower concentration than the other three groups. Sex differences were also reported in elapsed time processing with greater impairments observed in the female SHRs when discriminating longer time durations. These findings suggest a link between hippocampal function and ADHD behavioral symptomology, showing that SHRs exhibit working memory and elapsed time processing deficits similar to those reported in rats with hippocampus and medial entorhinal cortex (MEC) lesions (Sabariego et al., 2021; Vo et al., 2021). This study also introduced additional questions regarding the role of neuroinflammation in the observed SHR behavioral deficits.

In animal models of ADHD, using ecologically valid behavioral paradigms to explore the cognitive, behavioral, and neurobiological aspects of clinical conditions allows for better translation to human impairments observed in real-world settings. The traveling salesperson problem (TSP) is a complex yet naturalistic spatial navigational task that has been used to study route optimization, spatial memory, and spatial cognition in both human and non-human animals (Gärling, 1989; Wiener et al., 2007). In the rodent version of this task, rats navigate an arena consisting of a fixed array of targets, attempting to use the most efficient route to visit each target location once (Blaser, 2018). Prior studies have found that rats show spatial memory deficits on the TSP task following lesions of the hippocampus (Hales et al., 2021) and MEC (Hales et al., 2024). In contrast, lesions of the medial prefrontal cortex (mPFC) result in spatial decision-making, but not spatial memory, deficits (Kazanjian et al., manuscript in preparation). Given that the TSP task is naturalistic and hippocampus-dependent, it provides a valuable translatable paradigm for testing clinical models, such as the SHR model of ADHD.

Questions remain about ADHD pathogenesis, but recent studies have suggested a key role for neuroinflammation (Dunn et al., 2019). Microglia and cytokine expression levels have been used as biological markers for studying neuroinflammation. Specifically, microglia are the primary resident immune cells in the brain and serve as key mediators of neuroinflammation. They respond to injury or changes in the environment by releasing pro- or anti-inflammatory cytokines (Gao et al., 2023; Smith et al., 2012). In addition, the role that microglia play in the developing brain may contribute to developmental disorders (see Lenz and Nelson, 2018 for review).

To better understand the impairments in spatial working memory and decision-making in ADHD, this study examined the performance of male and female SHRs and WKY rats on the TSP task. Microglial activation was also assessed as a marker of neuroinflammation within the hippocampus, MEC, and prelimbic (PrL) and infralimbic (IL) regions of the mPFC, all of which are brain regions implicated in efficient spatial navigational performance on the TSP task. Microglia respond to insult or injury by altering their function and morphology, so in addition to measuring total microglial counts, this study quantified microglia in their different morphological states (such as ramified, amoeboid, and hypertrophic) to assess the degree and persistence of neuroinflammation in brain regions of interest.

## Methods

### Subjects

Subjects included 36 male and female SHRs and WKY rats (Charles River). There were 18 SHRs and 18 WKY rats, with equal numbers of each sex. Animals were pair housed and provided with ad libitum water access, but food access was regulated such that the rats were fed standard rat chow after testing each day and were food-deprived to 85% of ad libitum weight. The room was maintained on a 12/12 h light/dark cycle, and testing was performed during the light phase. Testing times were consistent within cohorts and across strains and sexes to control for performance differences due to time of day. Prior to testing, the rats were weighed and handled for 5 days, for 5 min per day. Rats were weighed and fed at the end of testing each day throughout TSP training and testing phases. The Institutional Animal Care and Use Committee (IACUC) at the University of San Diego approved all experimental protocols.

### Apparatus

The experimental apparatus included a circular open-field arena, 1 m in diameter, made of painted plywood. Flat plastic discs (2 in/5 cm diameter) were used as targets, with a single Cocoa Pebble™ placed on each target as bait. The arena was wiped down with a 50% ethanol solution after each trial and cleaned thoroughly between animals. All testing sessions were video recorded through a camcorder positioned directly above the arena.

### Test configurations

**Figure 1:**
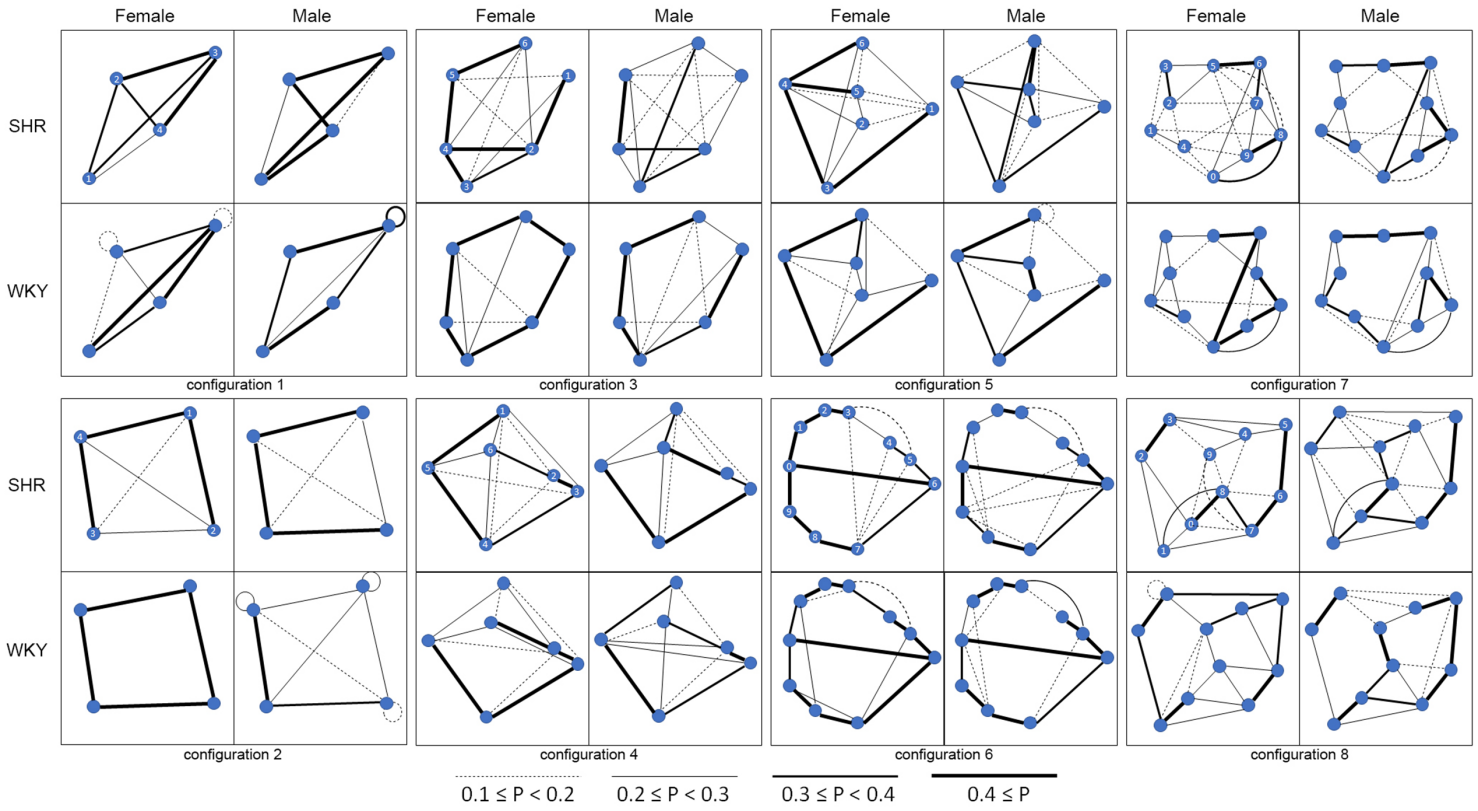
Each of the eight test configurations and the paths selected by the rats are illustrated. There are four boxes (2 × 2) for each configuration illustrating overall probability of making each inter-target transition by the SHR females (top left), SRH males (top right), WKY females (bottom left), and WKY males (bottom right). Legend at the bottom notes the relationship between line thickness and probabilities.

Rats were tested on eight configurations (Figure 1) in an open arena and were only tested on each configuration once. Configurations differed based on the number of targets and strategies that would produce the optimal route. Configurations 1 and 2 each had four targets and served as a baseline to ensure that rats were proficient in navigating the arena with a target layout that required a lower cognitive load. These two configurations were not assigned to a strategy type as they had too few targets to differentiate between strategies. Targets for Configurations 3 and 6 were located along the perimeter of the arena and the optimal route would be produced using the Hull strategy (Local-Perimeter, or L-P). This strategy involves rats navigating around the perimeter of the arena first and then working their way into the middle. Configurations 4 and 7 were similar, but differed in that a few targets were moved toward the middle of the arena, making the optimal strategy the Local-Nearest-Neighbor (L-NN) approach, in which the most optimal route is produced when rats navigate to the next closest target in the configuration. Configurations 5 and 8 focused on the Global Strategy, such that if rats used either of the previous two strategies, latency times would be significantly longer. Instead, rats need to take a big-picture approach to successfully navigate the configuration.

### Training behavioral procedure

The training phase lasted at least 9 days and continued until criterion was reached. Criterion was set at retrieval of nine out of 10 Cocoa Pebble™ targets for three consecutive days. On days one and two, rats were habituated to the open arena for 10 min. On the next day after habituation, four targets were placed in the arena; the number and position of targets varied each day, gradually increasing to 10 targets. Rats were required to eat the reward from all targets within 10 min before moving on to the next training level. Once rats achieved criterion, the testing phase began.

### Testing behavioral procedure

Testing in the TSP involved 8 days of being placed in the open-field arena, each day with a different configuration of baited targets (Figure 1). Every rat was tested on each configuration once in a pseudorandom order. All trials were timed and recorded with a stopwatch and a video camera for subsequent coding and analysis.

### Behavioral measures

After testing, all video files were manually coded by experimenters who were blind to sex and strain to record the sequence of target contacts. The coded variables included latency, contacts, total visits, revisits, omissions, and memory span. Latency was measured as the number of seconds taken to complete the task. Contacts were recorded when the rats’ whiskers, nose, or forepaws made contact with a target. Target retrieval was recorded when the rat removed the Cocoa Pebble™ from the target. From these values, the following behavioral measures were recorded. Revisits were visits to a target where the bait had already been retrieved. Transitions were the sequence of line segments connecting each successive target contact. The navigational paths were calculated for each group (by strain and sex) as the probability of making any given inter-target transition. Travel distance was the sum of those transition distances. Percent above optimal (PAO) was the difference between the actual travel distance and the distance of the optimal (most efficient) route for that configuration. Skips were visits to targets that did not result in the bait being retrieved. Memory span was the total number of consecutive target visits and bait retrievals before the first revisit. Rate was the total travel distance divided by the number of transitions, providing an average transition length. Proportion optimal transitions (PropOpt) was the proportion of the transitions made that fall along the optimal route. Proportion of distance on optimal route was the proportion of the total travel distance that fell along the optimal path. In addition, recorded trial videos were tracked using EthoVision software (Nodulus Information Technology) to obtain accurate measurements of travel velocity.

### Histological brain tissue processing

Once TSP testing was complete, rats were administered a fatal overdose of sodium pentobarbital and perfused transcardially with 0.01 M phosphate-buffered saline (PBS) followed by 4% paraformaldehyde (in 0.01 M PBS). Brains were removed from the skull and cryoprotected in 4% paraformaldehyde for 24 h, before being moved to 30% sucrose.

Twenty-four of the 36 rat brains (6 from each sex/strain) were processed for microglial expression. The other 12 rat brains (3 from each sex/strain) were sent to the Sabriego lab at Mount Holyoke College to be processed for cytokine expression (Anderson et al., 2024). The 24 brains were sectioned coronally with a freezing microtome at 40 μm. Every fifth section of the mPFC, hippocampus, and MEC was saved for microglial analysis. Immunolocalization of ionized calcium-binding adaptor molecule 1 (Iba-1, a protein marker for microglia) was performed using an anti-Iba-1, rabbit antibody (1:1,000, FijiFilm WACO; CAT#: 019-19741; 24 h rinse). A biotinylated goat anti-rabbit IgG (H + L) (1:200, Vector; CAT#: BA-1000-1.5; 2 h rinse) was used as the secondary antibody. Blocking solution consisted of normal goat serum (VWR; CAT#: 102643-594) in TritonX (Fisher; CAT#: BP151-100) and PBS. 3,3′-Diaminobenzidine (DAB) tablets (VWR; CAT#: E733-100tabs) were dissolved in PBS, with NiCl2-6H2O (0.3% of DAB solution) and 30% H2O2 (0.05% of DAB solution).

### Microglia cell counting

**Figure 2:**
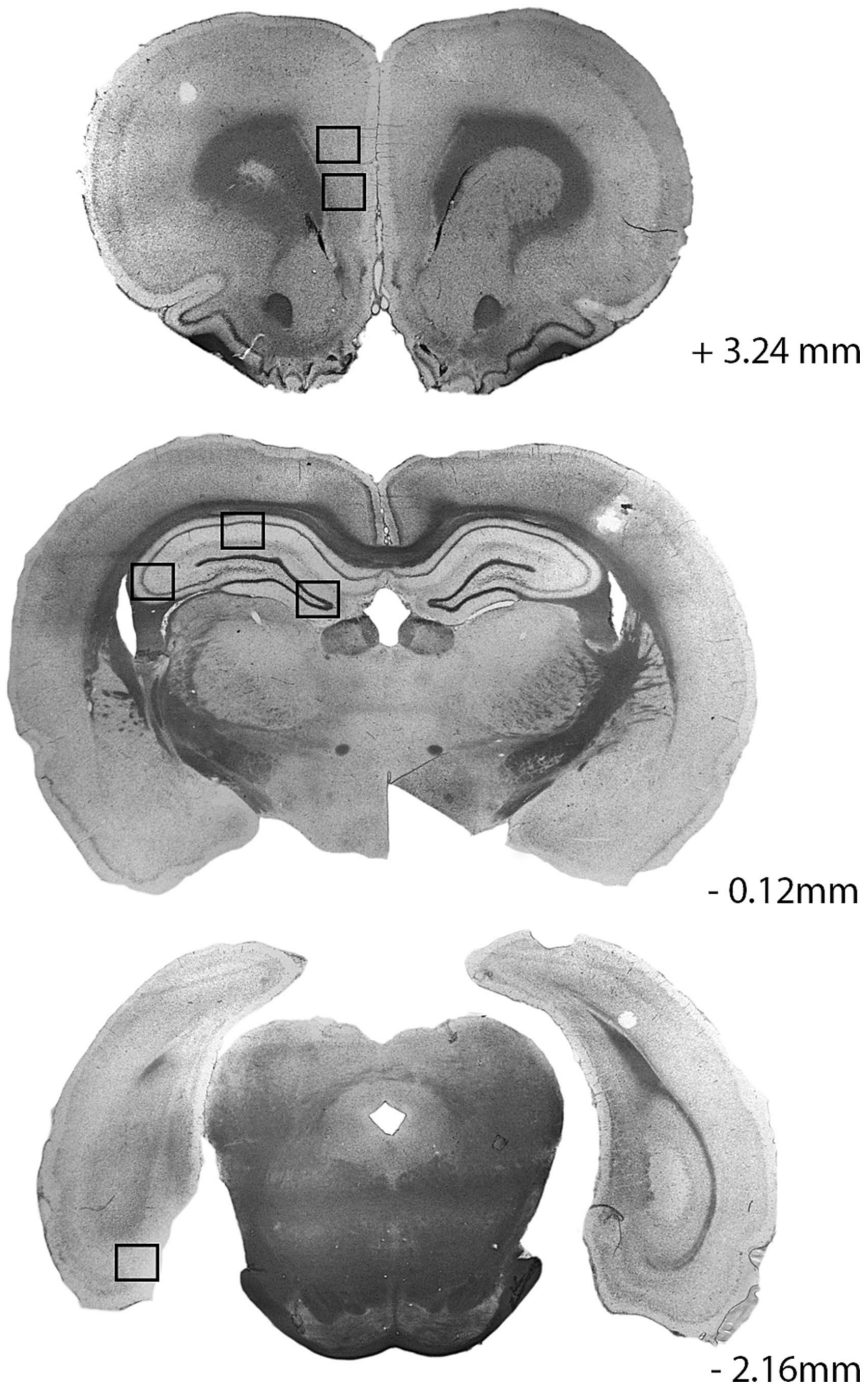
Brain regions of interest for microglial analyses shown on 40 μm coronal slices of tissue stained using immunolabeling of 1ba-1. Top to bottom: medial prefrontal cortex (mPFC) (+3.24 mm from Bregma), hippocampus (−0.12 mm from Bregma), and medial entorhinal cortex (MEC) (−2.16 mm from Bregma). Boxes represent subregions analyzed within the infralimbic (IL) and prelimbic (PrL) regions of the mPFC, the CA1, CA3, and dentate gyrus (DG) regions of the hippocampus, and the MEC.

**Figure 3:**
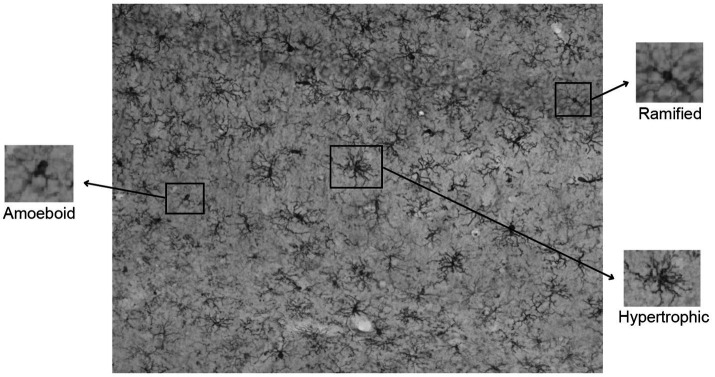
Differentiation and identification of different microglia morphologies (e.g., ramified, amoeboid, and hypertrophic). Example section of brain tissue within hippocampal CA1 following immunolabeling for 1ba-1.

Quantification of the microglia was performed using the Hybrid Cell Count program as part of the Keyence microscope software, on 20× magnification images taken from representative areas within the mPFC (PrL and IL regions), hippocampus (CA1, CA3, and dentate gyrus, DG), and MEC (Figure 2). The entire rectangular field of view within each region of interest (Figure 2), which includes layers with different neuronal and synaptic densities, was analyzed. These specific areas were chosen *a priori*, positioned centrally within each region of interest, and consistent across animals. After selecting these structural locations to analyze within each animal, a z-stack of images were taken from the bottom to the top cell layers spanning 1.5 μm apart. These images were then condensed and flattened into a single full-focus image. All photos were analyzed by the same experimenters (blind to the strain and sex) to ensure consistent quantification and classification across all brains. Microglial cells were first extracted by hue, selecting specifically for the darker cell bodies. If any processes or other dark stains were selected, they were deselected in the subsequent step to ensure accuracy and precision in the count. Once all of the microglial cell bodies were selected, the total number was noted, then differentiated between the amoeboid, ramified, and hypertrophic groups (Figure 3). The ramified microglia were identified first by selecting any microglia with circular cell bodies using the circularity histogram at a standardized upper limit of 1.03. The amoeboid microglia were then differentiated from the hypertrophic microglia by selecting those with fewer than three processes, signifying the amoeboid state. The remaining microglia, with larger cell bodies and more numerous and complex processes, were classified as hypertrophic. Brain sections with inconsistent staining were excluded from microglia quantification. Average percentages of hypertrophic microglia relative to the total microglial counts were calculated and reported for each sex, strain, and brain region of interest.

### Statistical analysis

All of the behavioral measures were calculated for each rat on each trial. Therefore, the experimental groups were compared using an Analysis of Variance (ANOVA), with L-P, L-NN, or Global configurations serving as within-subjects variables and the rat strain and sex serving as between-subjects variables. For each behavioral measure, this study compared SHR versus WKY and male versus female animals across the three categories of configurations (based on optimal search strategy) in a 2 × 2 × 3 repeated-measures ANOVA. A full summary of the results is presented in Table 1. For the microglia analyses, the experimental groups were compared using ANOVA, with the rat strain and sex serving as between-subjects variables.

**Table 1:** Summary of the results of the analysis of variance (ANOVA) for all behavioral measures.

Test	ME strain	ME sex	Strain × sex	Strain × strategy	Sex × strategy	Strain × sex × strategy
Revisits per target	ns	ns	ns	ns	ns	ns
Percent above optimal	ns	ns	ns	ns	ns	ns
Skips	ns	***p* = 0.028**	ns	ns	ns	ns
Span	ns	ns	ns	ns	ns	ns
Rate	***p* = 0.011**	ns	ns	ns	***p* = 0.018**	ns
Proportion optimal transitions	***p* = 0.034**	ns	***p* = 0.0496**	***p* = 0.031**	ns	ns
Proportion distance on optimal route	***p* = 0.0058**	ns	ns	***p* = 0.032**	ns	***p* = 0.044**
Velocity	***p* = 0.0093**	ns	ns	ns	ns	***p* = 0.037**

*p*-Values are reported for the main effects (ME) and Interactions (Int.) between strains (spontaneously hypertensive rats, SHR; and Wistar-Kyoto, WKY, rats), sexes, and all configurations varying by optimal strategy. Statistically significant effects are in bold.

## Results

### Behavioral measures

A full summary of the behavioral results is presented in Table 1.

Navigational paths: Figure 1 illustrates the probabilities of making inter-target transitions by rats in each group for each of the configurations.

**Figure 4:**
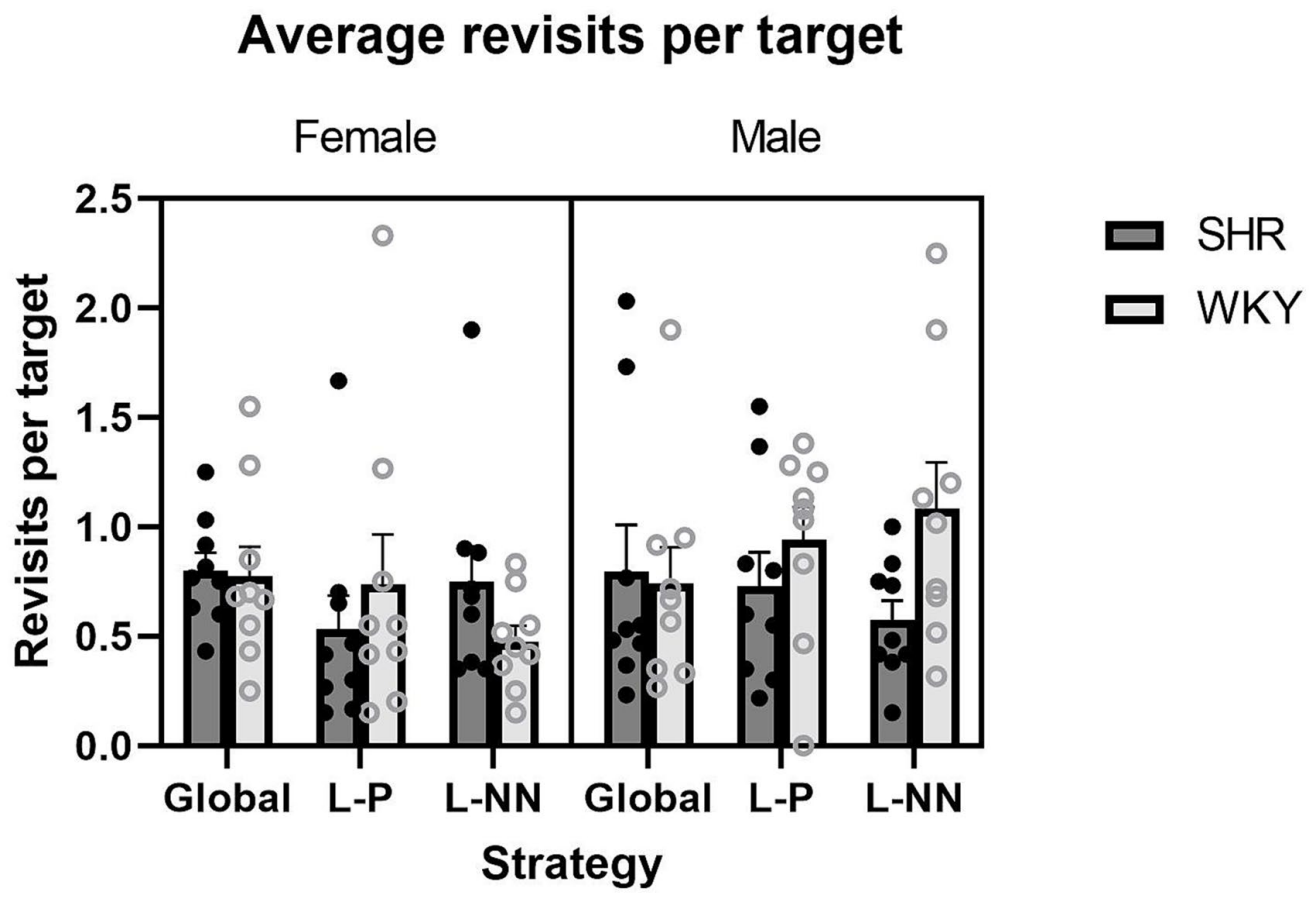
Average revisits per target is plotted as a function of strategy for SHR and WKY females (left) and males (right). No interactions or main effects of strain or sex were observed. Error bars represent standard error of the mean.

Revisits per target: ANOVA yielded no significant main effect of strain or sex on revisits per target. Therefore, there were no group differences in memory errors based on the number of times rats returned to targets where bait had already been retrieved. These results are illustrated in Figure 4.

Percent above optimal (PAO): ANOVA yielded no significant main effect of strain or sex on PAO indicating that the difference in actual distance traveled versus the distance of the optimal route for each configuration did not differ between groups.

**Figure 5:**
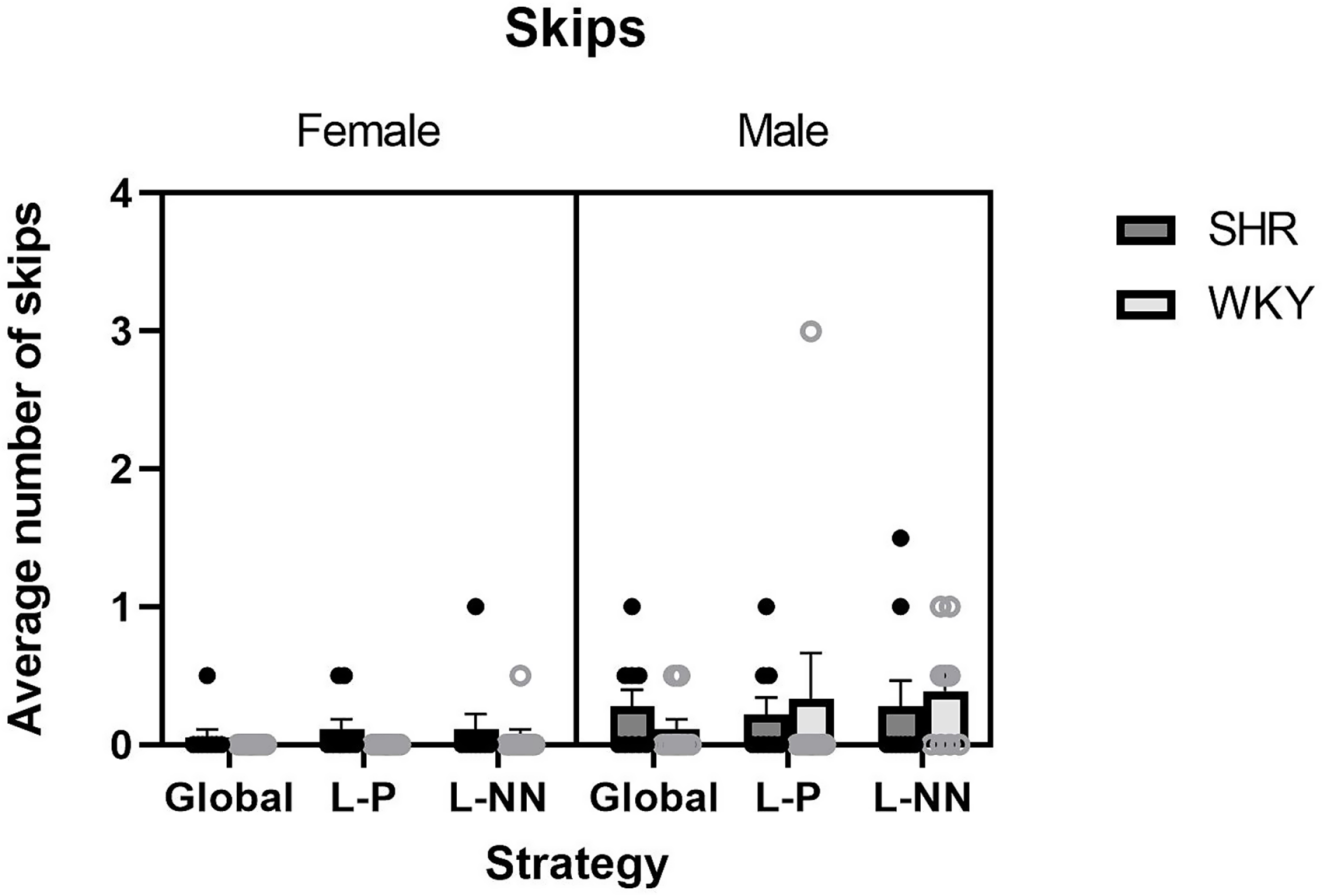
Average number of skips is plotted as a function of strategy for SHR and WKY females (left) and males (right). A main effect of sex was observed. Error bars represent standard error of the mean.

Skips: ANOVA yielded a significant main effect of sex (*F*(1, 16) = 5.86, *p* = 0.028), where males had more skips than females (*M* = 0.27 males and *M* = 0.06 females). There was no main effect of strain or any interactions. In other words, male rats (across both strains) made a greater number of visits to targets that did not result in the bait retrieval. These results are illustrated in Figure 5.

Span: ANOVA yielded no significant main effect of strain or sex on span indicating that there was no difference between the groups in the number of consecutive target visits and bait retrievals before the first revisit.

**Figure 6:**
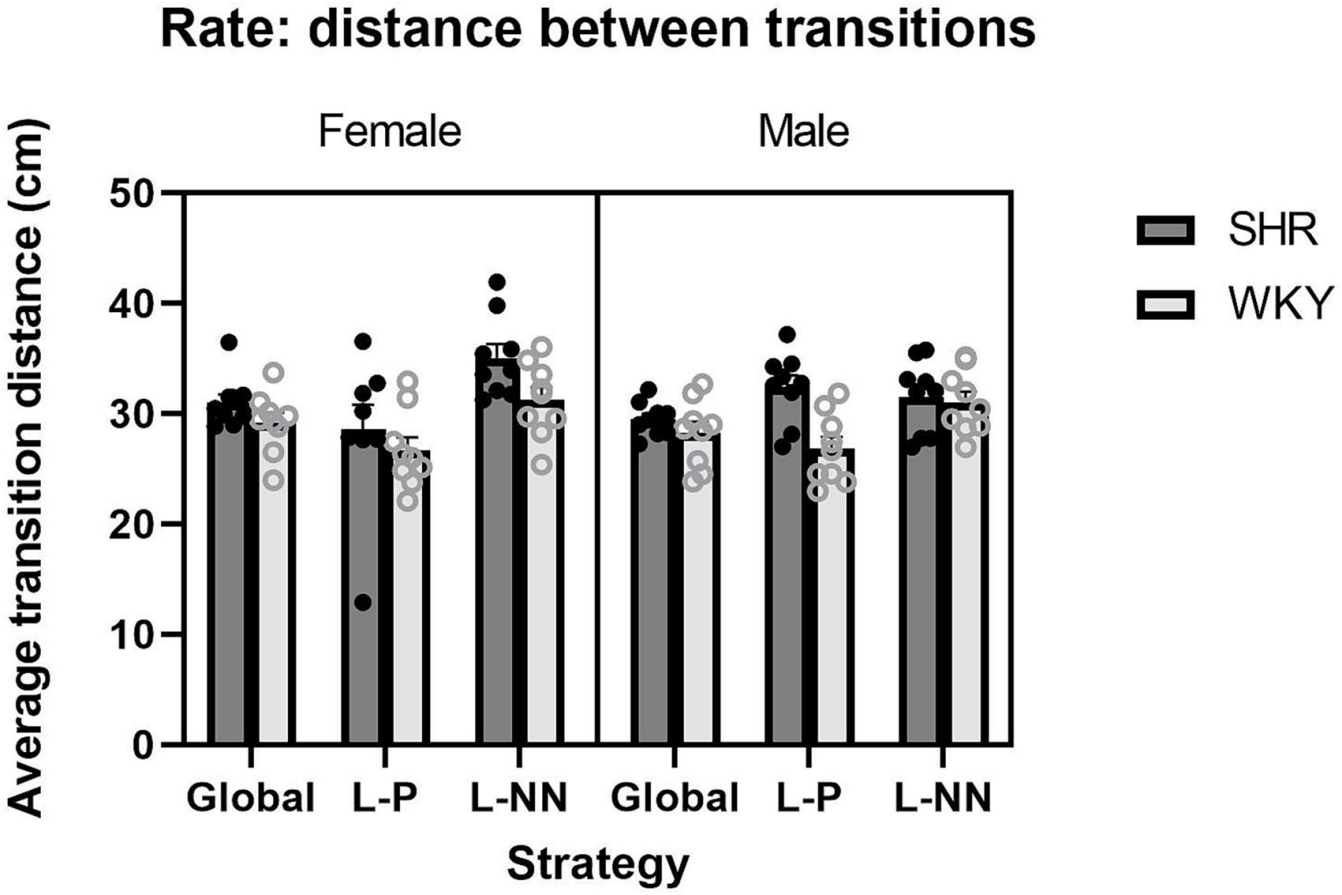
The average distance between transitions (“rate”) is plotted as a function of strategy for SHR and WKY females (left) and males (right). A main effect of strain was observed. A sex × strategy interaction was also observed. Error bars represent standard error of the mean.

Rate: ANOVA yielded a significant main effect of strain (*F*(1, 16) = 8.30, *p* = 0.011), with a greater distance between transitions for SHRs (*M* = 31.36 cm) than WKY rats (*M* = 28.88 cm). There was also a significant sex × strategy interaction (*F*(2, 32) = 4.55, *p* = 0.018), but no main effect of sex. Therefore, SHR rats traveled a greater average distance between successive target contacts than WKY rats. These results are illustrated in Figure 6.

**Figure 7:**
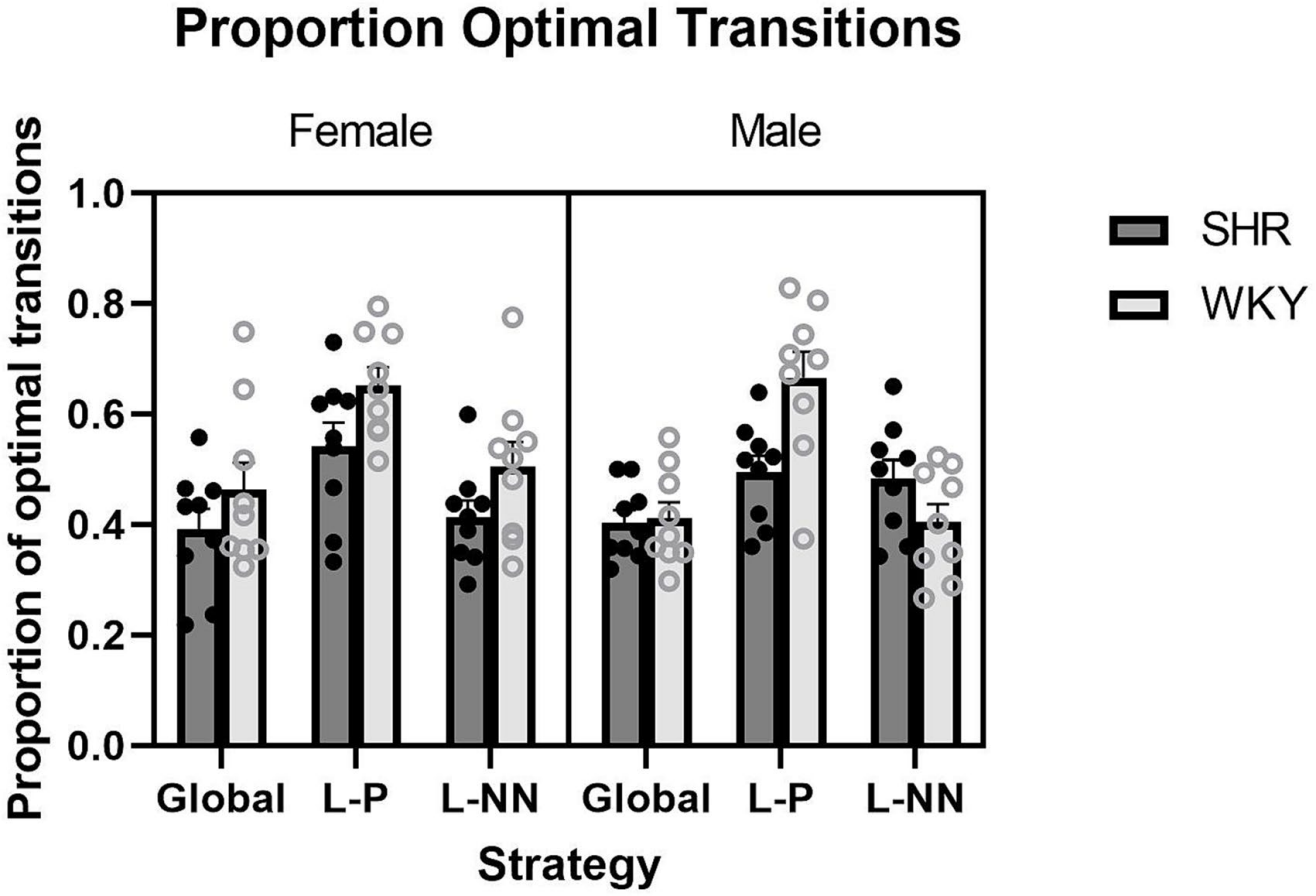
The proportion of optimal transitions (PropOpt) is plotted as a function of strategy for SHR and WKY females (left) and males (right). A main effect of strain was observed. A strain × sex interaction and a strain × strategy interaction were also observed. Error bars represent standard error of the mean.

Proportion optimal transitions (PropOpt): ANOVA yielded a significant main effect of strain (*F*(1, 16) = 5.41, *p* = 0.034), where WKY rats had a higher proportion of optimal transitions than SHRs (*M* = 0.52 WKY and *M* = 0.45 SHR). There was also a significant strain × sex interaction (*F*(1, 16) = 4.51, *p* < 0.05) and a significant strain × strategy interaction (*F*(2, 32) = 3.88, *p* = 0.031). In other words, SHRs made fewer transitions (successive target contacts) that fell along the optimal route than WKY rats. These results are illustrated in Figure 7.

**Figure 8:**
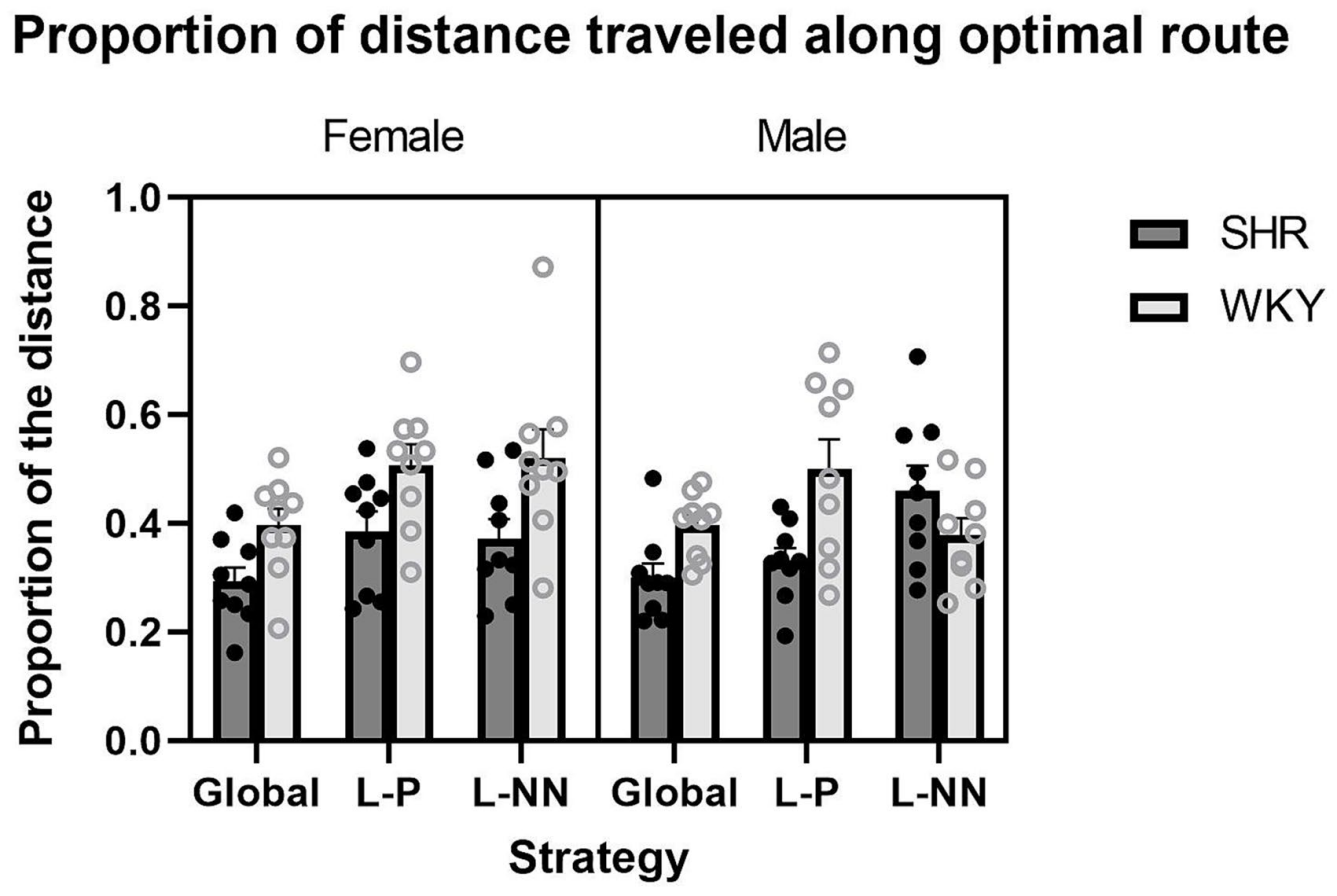
The proportion of distance traveled along the optimal route is plotted as a function of strategy for SHR and WKY females (left) and males (right). A main effect of strain was observed. A strain × strategy interaction and a strain × sex × strategy interaction were also observed. Error bars represent standard error of the mean.

Proportion of distance on optimal route: ANOVA yielded a significant main effect of strain (*F*(1, 16) = 10.11, *p* < 0.01), where a greater proportion of the distance was traveled along the optimal route in the WKY than SHRs (*M* = 0.45 WKY and *M* = 0.36 SHR). There was also a significant strain x × strategy interaction (*F*(2, 32) = 3.85, *p* = 0.032) and strain ×sex × strategy interaction (*F*(2, 32) = 3.44, *p* = 0.044). These results show that the SHRs spent less of their travel distance along the optimal route than the WKY rats. These results are illustrated in Figure 8.

**Figure 9:**
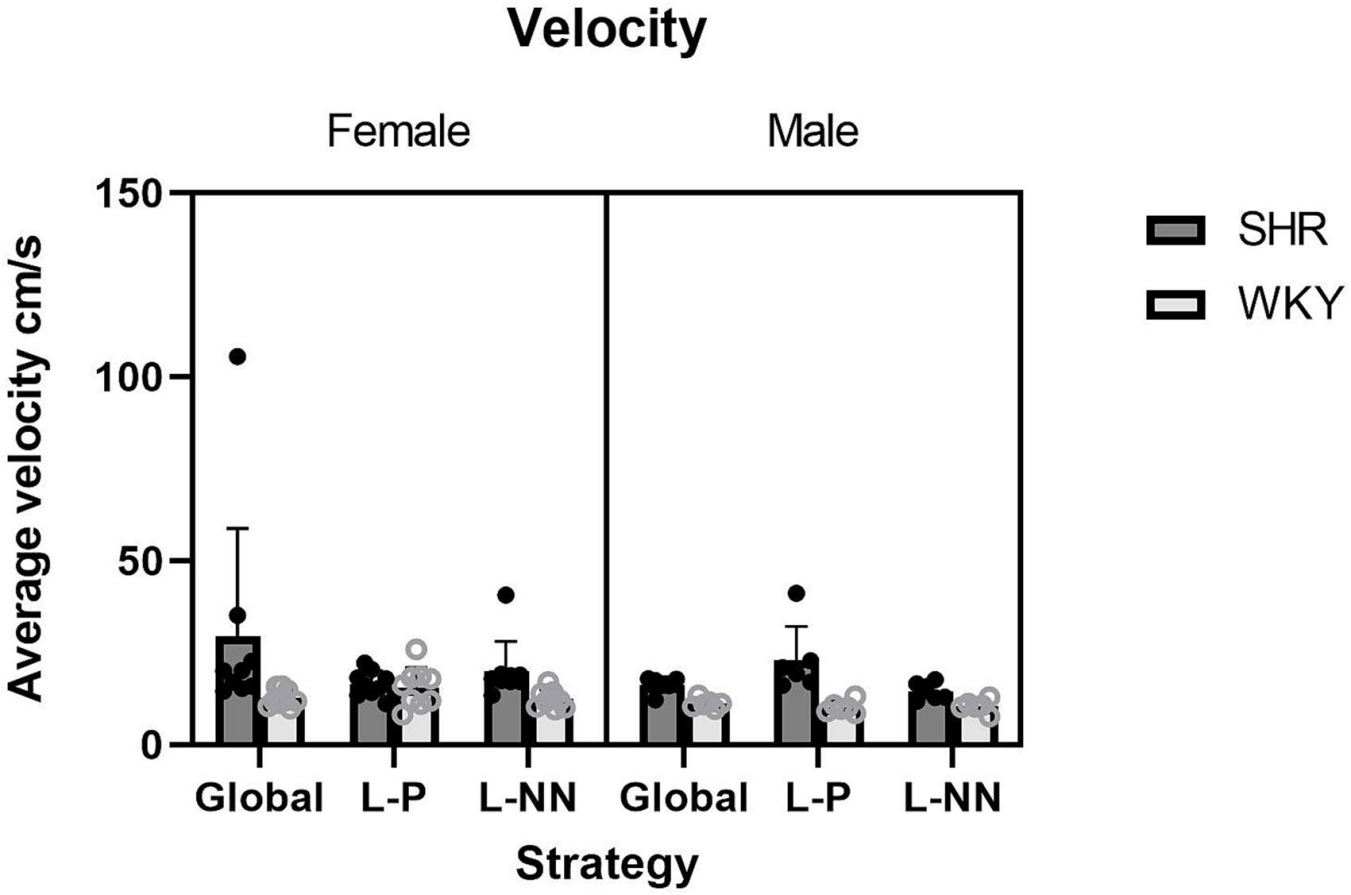
The average velocity is plotted as a function of strategy for SHR and WKY females (left) and males (right). A main effect of strain was observed. A strain × sex × strategy interaction was also observed. Error bars represent standard error of the mean.

Velocity: The mixed effects analysis (due to missing values) yielded a significant main effect on strain (*F*(1, 26) = 7.90, *p* < 0.01), with a greater average velocity in the SHRs than WKY rats (*M* = 20.43 cm/s SHR and *M* = 12.40 cm/s WKY). There was also a significant strain × sex × strategy interaction (*F*(2, 32) = 4.18, *p* = 0.024). These results are illustrated in Figure 9.

### Microglia cell counting

After excluding tissue with inconsistent staining from microglia quantification, the total number of brains included in the microglial counts within each brain region were: PrL/IL (*n* = 17 total; 4 female SHR, 4 male SHR, 3 female WKY, and 6 male WKY), hippocampus (*n* = 19; 5 female SHR, 4 male SHR, 6 female WKY, and 4 male WKY), and MEC (*n* = 14; 5 female SHR, 2 male SHR, 4 female WKY, and 3 male WKY).

**Figure 10:**
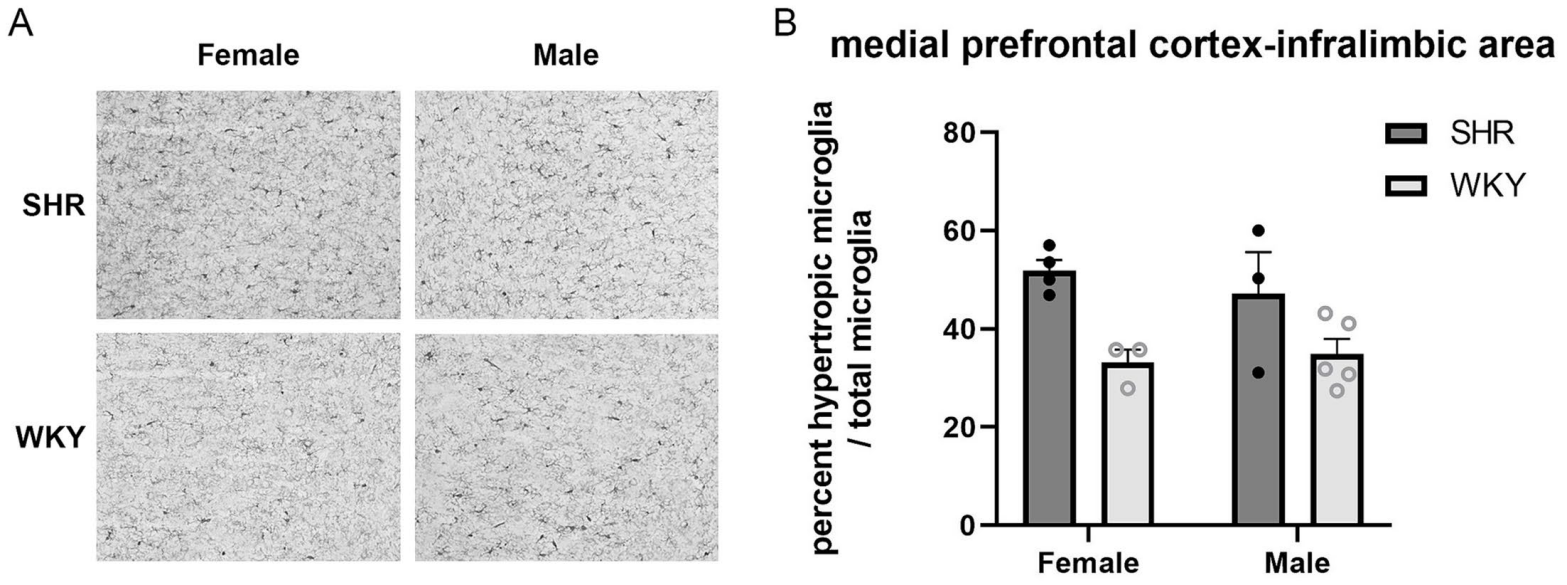
Larger percentage of hypertrophic microglia in the SHR than WKY group within the infralimbic (IL) region of the medial prefrontal cortex (mPFC). **(A)** Representative images of Iba-1 immunolabeled microglia in the IL in female and male SHR and WKY rats. **(B)** Percent hypertrophic microglia relative to the total number of microglia within the IL region of the mPFC plotted for SHR and WKY female and male rats. A main effect of strain was observed. Error bars represent standard error of the mean.

**Figure 11:**
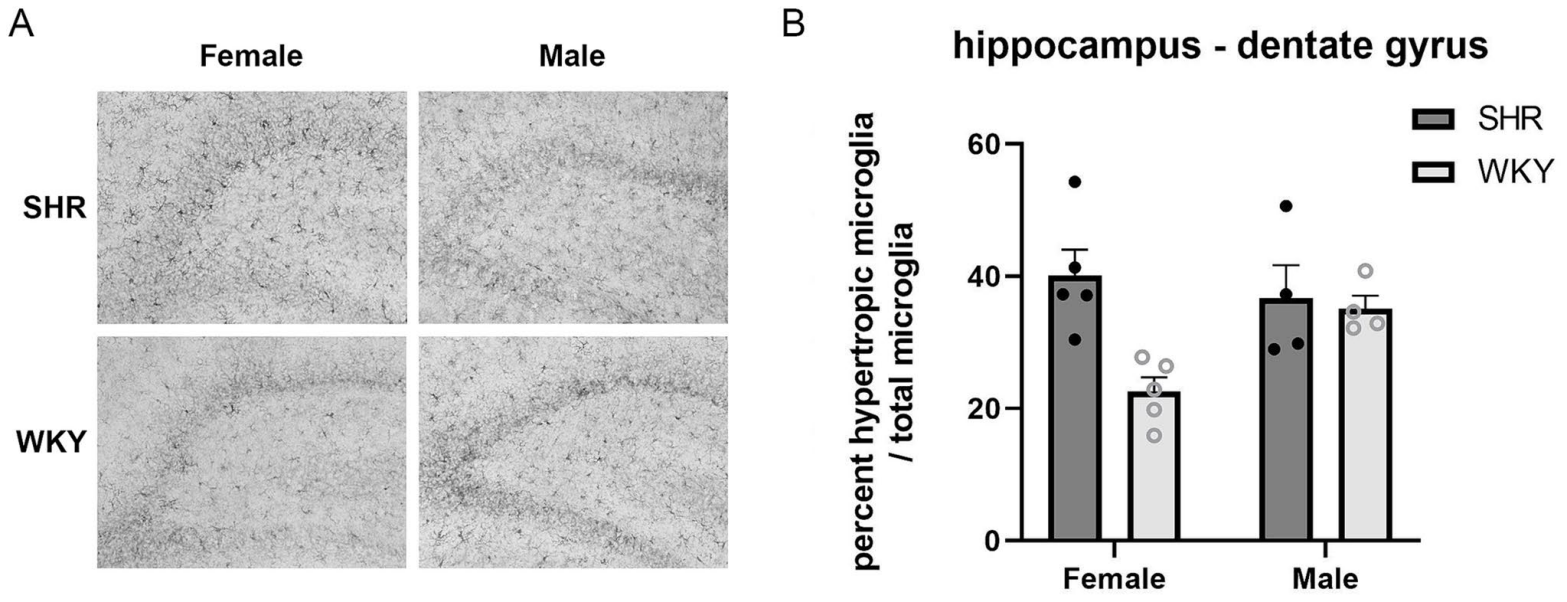
Larger percentage of hypertrophic microglia in the SHR females than the WKY females within the dentate gyrus region of the hippocampus. **(A)** Representative images of Iba-1 immunolabeled microglia in the dentate gyrus in female and male SHR and WKY rats. **(B)** Percent hypertrophic microglia relative to the total number of microglia within the dentate gyrus region of the hippocampus plotted for SHR and WKY female and male rats. A main effect of strain was observed. A strain × sex interaction was also observed. Error bars represent standard error of the mean.

There were no main effects of sex found in any of the brain regions. In the IL region of the mPFC, there was a main effect of strain (*F* (1, 11) = 13.12, *p* < 0.01), with a larger percentage of hypertrophic microglia in the SHRs than WKY rats (*M* = 49.84% SHR and *M* = 34.23% WKY). These results are illustrated in Figure 10. There was no main effect of strain in the PrL region of the mPFC. In the hippocampus, there was a main effect of strain in the dentate gyrus (*F* (1, 14) = 7.50, *p* = 0.016), with a larger percentage of hypertrophic microglia in the SHRs than WKY rats (*M* = 38.56% SHR and *M* = 28.15% WKY). There was also a strain × sex interaction (*F*(1, 14) = 5.26, *p* = 0.038), with females showing a larger percentage of hypertrophic microglia in SHR than WKY. These results are illustrated in Figure 11. There were no main effects of strain in the CA1 or CA3 regions of the hippocampus or in the MEC. A full summary of the microglia analyses is presented in Table 2.

**Table 2:** Summary of the results of the analysis of variance (ANOVA) for all microglia measures (percent hypertrophic microglia/total microglia) for each brain region of interest.

Brain region	ME strain	ME sex	Strain × sex
Medial prefrontal cortex—prelimbic (PrL)	ns	ns	ns
Medial prefrontal cortex—infralimbic (IL)	***p* = 0.004**	ns	ns
Hippocampus—CA1	ns	ns	ns
Hippocampus—CA3	ns	ns	ns
Hippocampus—dentage gyrus (DG)	***p* = 0.016**	ns	***p* = 0.038**
Medial entorhinal cortex (MEC)	ns	ns	ns

*p*-Values are reported for the main effects (ME) and interactions (Int.) between strains (spontaneously hypertensive rats, SHR; and Wistar-Kyoto, WKY, rats) and sexes. Statistically significant effects are in bold.

## Discussion

The TSP task is a cognitively-rich, ecologically valid behavioral paradigm with translational value for research involving animal models under clinical conditions (De Vreese et al., 2005). In this study, naturalistic spatial foraging behavior was examined in the ADHD-model SHRs versus WKY control rats to measure differences in spatial memory versus route-selection and decision-making processing. Spatial memory and decision-making are important cognitive functions that help individuals navigate their everyday lives. Such processing, however, can be more challenging with certain neurological conditions, including ADHD (Del Lucchese et al., 2021). By isolating and measuring different aspects of cognitive processing that contribute to natural behavior, this study may directly examine the differences between female and male SHR and WKY rat behavior, elucidating possible sex-based differences in ADHD symptomology. Finally, using animal models of clinical conditions allows us to examine both behavior and pathology in the same animals and continue exploring the links between neuroinflammation and behavioral impairments.

The results demonstrate that ADHD-model rats, the SHR group, perform less efficiently on the TSP task than the WKY control rats. The TSP task allows for measuring multiple cognitive processes. As previously discussed in Hales et al. (2021), manipulating target number and optimal strategy can reveal the underlying mechanisms of cognitive processes. Measures that covary with increasing target number (such as revisits per target) are indicators of spatial memory, whereas measures that covary with strategy (such as proportion of the distance along the optimal path) are indicators of route selection and decision-making. The SHR group did not show a difference in number of revisits per target or memory span, suggesting intact spatial memory performance. In contrast, the SHR group was impaired on the measures of route selection and spatial decision-making, such as the proportion of distance traveled along the optimal route, the proportion of optimal transitions, and the rate. In all three measures, the WKY rats performed more efficiently: greater proportion of distance along the optimal route, greater proportion of transitions along the optimal route, and less distance traveled between transitions. Additionally, the SHRs had a greater average velocity than the WKY rats, which likely represents the hyperactivity generally seen in the SHR strain (Fasmer and Johansen, 2016). Moving around the open field with a greater velocity can lead to less efficient sampling of the spatial environment and less optimal route selection. Based on all of these findings, this study conclude that SHR have deficits in spatial decision-making and route-selection but not spatial memory.

The spatial configurations used in this study were designed so that certain exploration strategies would result in more optimal paths than others. The Local-Nearest-Neighbor (L-NN) approach (optimal for Configurations 4 and 7) relies on using local cues, whereas the Hull strategy (L-P) (optimal for Configurations 3 and 6) involves using a stereotyped circling approach. Finally, a more Global strategy (optimal for Configurations 5 and 8) is necessary when the L-NN and L-P approaches alone would result in inefficient paths. In the behavioral analyses, performance was separated by configurations based on the optimal strategies in order to examine whether SHRs used similar or different exploration strategies. Both the proportion of optimal transitions (PropOpt) and proportion of distance traveled along the optimal route found a strain × strategy interaction, with WKY rats performing better than (or the same as) the SHRs on all strategies, with the exception of male SHRs performing better than male WKY rats only on the L-NN configurations. These results suggest that the male SHRs may have relied more heavily on local cues to navigate efficiently.

The pattern of deficits seen in the SHR group was distinct from those previously reported in rats following hippocampal lesions (Hales et al., 2021) or MEC lesions (Hales et al., 2024). Hippocampus- and MEC-lesioned rats were impaired on measures of spatial memory, showing more revisits per target than in the sham-lesioned controls, and hippocampus-lesioned rats also showed an impairment with memory span. Neither measure showed a main effect of strain in the current study. Measures of route selection and spatial decision-making, however, were not affected by hippocampus or MEC lesions, as both lesioned and sham groups performed similarly on measures of proportion of optimal transitions (PropOpt) and proportion of distance traveled along the optimal route. Such results are in contrast to the present study in which the SHR group was impaired on these measures relative to the WKY rats. Both lesion groups had longer pathlengths (PAO) than their control groups, due to the increased number of revisits. In this study, no main effect of strain or sex for PAO. Hippocampus-lesioned rats, but not MEC-lesioned rats, were impaired on their average transition distance (“rate”), which was also seen in the SHR group. Since hippocampus-lesioned rats were impaired on rate, but not the other measures of route selection/decision-making, the researchers suggested this may represent difficulty with using local cues to guide their decisions (Hales et al., 2021). On the other hand, SHRs were impaired on multiple measures of route selection, suggesting a broader spatial decision-making deficit.

The only TSP measure with a main effect of sex was the number of skips, with males making more skips than females. Previous lesion studies found an increased number of skips in hippocampus-lesioned rats, but not MEC-lesioned rats. The researchers interpreted this deficit as representing a general impairment in attention or motivation in hippocampus-lesioned rats. Findings in this study suggest a possible sex difference in general attention or motivation on the TSP that is independent of strain. Finally, similar to the findings of a greater average velocity in the SHR group, MEC-lesioned rats had a greater average speed than the control group (speed/velocity was not reported in the hippocampus-lesion study). It is relevant to note that unpublished work from the lab examined the effects of PrL/IL lesions on TSP performance in rats. These findings are similar to those reported in the SHR group and distinct from the impairments reported in hippocampus- and MEC-lesioned rats, as PrL/IL-lesioned rats showed deficits in route selection measures, but not in measurement of spatial memory (Kazanjian et al., manuscript in preparation). Previous studies examining the effect of PrL/IL lesions on spatial memory have also reported intact spatial memory decisions, but impaired performance when rats had to use cognitive flexibility (Park et al., 2025) and adapt their behavioral responses flexibly to new conditions (Gisquet-Verrier and Delatour, 2006). Decreased cognitive and behavioral flexibility may contribute to inefficient route selection in the TSP.

Findings from this study expand on study by Anderson et al. (2024), which examined working and elapsed-time memory in male and female SHRs and WKY rats and measured cytokine levels in the hippocampus. On the delayed alternation task, SHRs showed a delay-dependent working memory deficit that was similar to, but more moderate than, deficits observed in rats with hippocampal lesions. On the time duration discrimination task, female SHRs were impaired in discriminating longer time delays, similar to deficits previously reported in rats with hippocampal lesions (Sabariego et al., 2021) and MEC lesions (Vo et al., 2021). These findings comparing the behavioral impairments between SHRs and rats with hippocampus or MEC lesions, suggest that medial temporal lobe function may contribute to the SHR behavioral deficits (Anderson et al., 2024). In contrast, this present study using the TSP task, however, did not find memory deficits in the SHR group, as measured by revisits and memory span. Given that the SHR memory deficits reported in Anderson et al. (2024) were more minor than those seen with hippocampal lesions, it is possible that the TSP task did not produce a large enough demand on working memory capacity to show an SHR deficit.

Although the hippocampal cytokine measures in Anderson et al. (2024) only found mild differences in expression between male and female SHRs and WKY rats and did not provide evidence of strain differences, the authors noted that the age of the rats at sacrifice (12+ weeks) could be a possible explanation for the lack of expression differences. Based on previous studies that have reported elevated levels of cytokines (including IL-1β in serum and IL-1β, IL-6, and TNF-ɑ) in SHRs at 5 weeks of age (Kozłowska et al., 2019), neuroinflammation and elevated cytokine expression still could have occurred in early developmental stages in SHRs but then normalized by the time of sacrifice and cytokine measurement. Therefore, early neurodevelopmental inflammatory responses and cytokine expression in SHR may contribute to later behavioral changes (Anderson et al., 2024). This study examined a different marker of neuroinflammation, specifically for measuring microglial expression in the brain regions of interest.

Microglia and their role in neuroinflammation have become an area of focus in ADHD research (Bordeleau et al., 2019). Microglia have several states of activation, including amoeboid (immature), ramified (mature/healthy), and hypertrophic (reactive) (Calcia et al., 2016). Microglia can be further classified into physiological and pathological sub-categories (Augusto-Oliveira et al., 2025). Within the physiological subcategory, microglia can be classified as surveilling or satellite. Pathological morphotypes include hyper-ramified, hypertrophic, amoeboid, dystrophic, dark, and rod-shaped. Given the structural similarities between many of these morphotypes, it can be difficult to accurately distinguish between them. For the purpose of this study, classical morphotypes were used, including hyper-ramified, surveilling, amoeboid, and hypertrophic. Hyper-ramified and surveilling morphotypes were further combined into a singular ramified category due to structural similarities. Amoeboid microglia are common in early development, while ramified microglia are associated with a more mature, healthy, resting-state, and they monitor the environment and screen the central nervous system for infection and damage. When ramified microglia respond to an insult or injury, the soma enlarges and processes retract, taking on an amoeboid morphology. When microglia are activated for long periods of time, in the case of chronic inflammation, they become hypertrophic and increase their release of pro-inflammatory cytokines. Recent studies have shown that microglial morphology, function, and quantity are altered by a variety of neurodevelopmental disorders, including ADHD (Yokokura et al., 2021; see Lukens and Eyo, 2022). Microglia play a critical role not only in neuroinflammation, but also in synaptic pruning, circuit refinement, and other neurodevelopmental processes (Dunn et al., 2019). Altered microglial states during development can result in impaired functioning of the hippocampus (Crum et al., 2017; Fang et al., 2023; Piontkewitz et al., 2012; Sterley et al., 2013) and PFC (Breach et al., 2022; Fang et al., 2023; Arnsten and Rubia, 2012; Lyamtsev et al., 2025).

Results from this study support previous findings, as both a larger percentage of hypertrophic microglia in subregions of the hippocampus and mPFC, and behavioral impairments in route selection and spatial decision-making, were observed in SHRs compared to WKY rats. It is important to note that this study does not examine causation between neuroinflammation and behavioral performance; therefore, other possible explanations for these two effects, including hyperactive behavior increasing neuroinflammatory responses or a third factor contributing to both. Although there is limited research examining the role of microglia activation in the IL region of the mPFC in ADHD, a recent study found increased levels of Iba-1 in the IL/PrL and pro-inflammatory transcription factor NF-kB in the IL in perinatal nicotine-exposed mice (Uygun et al., 2025). Notably, prenatal nicotine exposure has been associated with childhood ADHD (Gustavson et al., 2017). In this study, the SHR group also had a higher percentage of hypertrophic microglia in the dentate gyrus of the hippocampus. Additionally, there was an interaction between sex and strain, with female SHRs showing a greater percentage of hypertrophic microglia than female WKY rats in the dentate gyrus, with no significant difference between strains for the male rats. This significant difference in microglia activation in the dentate gyrus of the female SHRs provides evidence of a sex-based difference in neuroinflammation and further emphasizes the importance of examining sex as a biological variable. It is interesting that greater neuroinflammation was observed in the dentate gyrus of SHRs, but no difference was found in the TSP measures of memory that were impaired in previous studies of rats with hippocampal lesions (Hales et al., 2021). However, unlike these lesions that involved damage to the entire hippocampus including all cell fields, this study found differences in microglia activation in the dentate gyrus and not in the CA fields of the hippocampus. This finding suggests that either the dentate gyrus of the hippocampus is involved in different aspects of TSP behavior or inflammation in the dentate gyrus may not be severe enough to disrupt spatial memory on the TSP task. Different behavioral tasks place various amounts of burden on memory systems. Tightly controlled, manipulated experimental paradigms can be designed to produce greater demands on memory, such as the delayed alternation task and time-duration discrimination task. More naturalistic tasks, such as the novel object recognition task and the TSP task, involve the observation of natural behaviors with lesser demands on memory systems. These more passive tasks often show more subtle memory impairments following hippocampal damage (Hales et al., 2021; see Clark and Martin, 2005 for review). Taken together, these findings suggest that neuroinflammation in the dentate gyrus of SHRs may be sufficient to disrupt memory performance on more demanding memory tasks, such as those examined in Anderson et al. (2024), but not on the less demanding TSP task.

Targeting the microglial neuroinflammatory response has been examined as a possible route for therapeutic intervention. Methylphenidate (MPH), also known as Ritalin, is a common treatment for ADHD. MPH treatment at a clinically relevant dose in SHRs was found to attenuate inflammation by altering microglia morphology from an activated to resting state and improve ADHD-like symptoms such as hyperactivity (Coelho-Santos et al., 2018). Notably, the findings were reversed at a higher dose of MPH in the SHR group and at both low and high doses of MPH in the WKY group (Coelho-Santos et al., 2018) and in Sprague Dawley rats (Carias et al., 2018). These findings suggest that MPH acts on microglia in a dose- and strain-dependent manner. Another common treatment for ADHD, lisadexamfetamine (LDX), had similar effects on microglial expression in control rats, increasing microglial activation in the hippocampus and impairing spatial memory (Nourirad et al., 2024). Another recent study found that inhibiting prefrontal microglial activation and neuroinflammation using a novel traditional Chinese herbal formulation (Bushen Kaiqiao Formula) reduced the behavioral deficits in SHRs (Zhang et al., 2025). Taken together, these studies provide evidence that inhibiting microglial activation may help improve ADHD symptomology.

Gender- and sex-biases have historically directed clinical and preclinical animal research toward male subjects, which has optimized the understanding of human diseases, diagnoses, and treatments for biological males. In an effort to address this bias, the National Institutes of Health in 2016 began requiring all grant applicants to explicitly describe how their study would consider sex as a biological variable, but a lack of accountability and real commitment to change has persisted across areas of science (Garcia-Sifuentes and Maney, 2021). This study directly examined sex as a biological variable in both the behavioral and histological analyses. For the microglia analyses, there were no main effects of sex, but a sex-by-strain interaction was observed, due to a larger percentage of hypertrophic microglia in the dentate gyrus of female SHRs compared to female WKY rats. For the behavioral analyses, some significant interactions with sex were found, but the only main effect of sex was for skips, with males showing more skips than females. Although only minor sex-based differences were identified in this study, it remains critical for scientists to conduct research in ways that allows for analysis and exploration of sex as a biological variable. Despite recognition of sex-based discrepancies in neurological disorders, ADHD research in humans has also heavily relied on male subjects. Evidence of the lack of biological females included in ADHD research can be seen in diagnosis rates, as biological males are more than twice as likely to be diagnosed with ADHD compared to biological females (13% vs. 6%; reported by the Center for Disease Control, 2024). The limited empirical data surrounding sex differences and factors underlying disease progression has also negatively impacted the treatment and care of female patients with ADHD (Young et al., 2020). Therefore, there is a strong need to better understand the neurobiological mechanisms and systems underlying sex-related differences in ADHD. Findings from this study suggest potential sex differences in both hippocampal neuroinflammation and TSP behavior in SHRs that warrant further exploration.

In conclusion, the study results demonstrate clear deficits in SHR performance on the TSP task, specifically on measures related to route selection and spatial decision-making. They were not impaired; however, on measures of spatial memory. These findings contrast with the deficits previously found in rats with hippocampus- or MEC-lesions, but are consistent with deficits observed in rats with mPFC-lesions. This study also found that male and female SHRs showed similar deficits, with male rats only performing worse on a measure of attention or motivation. Given the translational value of the TSP task, these behavioral findings suggest that the SHR rodent model of ADHD displays navigational deficits due to impairments in route selection and not spatial memory. The findings of increased hypertrophic microglia expression in subregions of the mPFC and hippocampus provide evidence of chronic neuroinflammation in SHRs and demonstrate a possible link between the neurobiological changes and behavioral deficits. Future studies should examine whether directly targeting these microglia neuroinflammatory responses alleviates TSP performance deficits in female and male SHRs. In addition, researchers using animal models of ADHD should continue to use naturalistic paradigms, such as the TSP task, to ensure findings more directly translate into understanding real-world disease symptomology. It is also critical for researchers to intentionally examine sex as a biological variable to gain insights into the neurobiological foundations of sex-related differences in ADHD.

## Data Availability

The raw data supporting the conclusions of this article will be made available by the authors without undue reservation.
